# Thermophoresis: The Case of Streptavidin and Biotin

**DOI:** 10.3390/polym12020376

**Published:** 2020-02-07

**Authors:** Doreen Niether, Mona Sarter, Bernd W. Koenig, Jörg Fitter, Andreas M. Stadler, Simone Wiegand

**Affiliations:** 1 Institute of Biological Information Processing (IBI-4: Biomacromolecular Systems and Processes) & JARA-SOFT, Forschungszentrum Jülich GmbH, D-52428 Jülich, Germany; 2 I. Physikalisches Institut (IA) & JARA-SOFT, AG Biophysik, RWTH Aachen, Sommerfeldstrasse 14, 52074 Aachen, Germany; mona.sarter@stfc.ac.uk (M.S.); fitter@physik.rwth-aachen.de (J.F.); 3 Jülich Centre for Neutron Science (JCNS-1) and Institute of Biological Information Processing (IBI-8: Neutron Scattering and Soft Matter), Forschungszentrum Jülich GmbH, D-52428 Jülich, Germany; a.stadler@fz-juelich.de; 4 Institute of Biological Information Processing (IBI-7: Structural Biochemistry), Forschungszentrum Jülich GmbH, D-52428 Jülich, Germany; b.koenig@fz-juelich.de; 5 Institute of Biological Information Processing (IBI-6: Molecular Biophysics), Forschungszentrum Jülich GmbH, D-52428 Jülich, Germany; 6 Institute of Physical Chemistry, RWTH Aachen University, Landoltweg 2, D-52056 Aachen, Germany; 7 Department für Chemie—Physikalische Chemie, Universität zu Köln, 50939 Cologne, Germany

**Keywords:** thermophoresis, protein–ligand binding, hydrogen bonds

## Abstract

Thermophoretic behavior of a free protein changes upon ligand binding and gives access to information on the binding constants. The Soret effect has also been proven to be a promising tool to gain information on the hydration layer, as the temperature dependence of the thermodiffusion behavior is sensitive to solute–solvent interactions. In this work, we perform systematic thermophoretic measurements of the protein streptavidin (STV) and of the complex STV with biotin (B) using thermal diffusion forced Rayleigh scattering (TDFRS). Our experiments show that the temperature sensitivity of the Soret coefficient is reduced for the complex compared to the free protein. We discuss our data in comparison with recent quasi-elastic neutron scattering (QENS) measurements. As the QENS measurement has been performed in heavy water, we perform additional measurements in water/heavy water mixtures. Finally, we also elucidate the challenges arising from the quantiative thermophoretic study of complex multicomponent systems such as protein solutions.

## 1. Introduction

Especially in recent years, thermophoresis, which is the mass transport caused by a temperature gradient, has gained a lot of interest [1]. Two subject areas have mainly contributed to this research boost especially in aqueous solutions of biological and biocompatible compounds: on the one hand, the accumulation of molecules in thermophoretic traps, by a combination of thermodiffusion and convection [2,3], and, on the other, the detection of binding reactions via the change in the thermophoresis of a protein when a ligand binds. The first topic is of great interest as an enrichment process for prebiotic molecules in the context of theories on the ‘origin-of-life’. The second observation is used in biophysics and biomedical research to study binding constants of pharmaceutical compounds [4].

The thermophoresis of molecules is influenced by several factors like temperature, concentration, ionic strength, mass, and moment of inertia [1,5,6]. Another important factor is the interaction of a molecule or a colloid with the surrounding solvent. This contribution has significant influence on thermodiffusion in polar solvents and is expected to be relevant for biomolecules in aqueous solutions, where interactions are dominated by hydrogen bonds [7,8,9]. It has been observed that thermodiffusion of biomolecules changes strongly when conformational changes occur, for example due to a ligand binding to a protein [10] or due to protein unfolding [11]. This is often explained with structural changes in the hydration shell [3,9,12]. Microscale thermophoresis (MST) exploits this sensitivity of thermophoresis to conformational changes to measure equilibrium binding constants [4].

The amplitude of the concentration gradient is determined by the Soret coefficient $S_{T}$. For the temperature dependence of $S_{T}$ in aqueous solutions, an empirical equation was proposed by Iacopini and Piazza [13],

(1) $$S_{T}\left(T\right)=S_{T}^{\infty }\left[1-\exp\left(\frac{T^{*}-T}{T_{0}}\right)\right]\;,$$

where $S_{T}^{\infty }$ is a constant value that is approached at high temperatures, $T^{*}$ is the temperature of the sign change and $T_{0}$ characterizes the slope. This equation describes many diluted solutions of water soluble solutes well: the Soret coefficient is low, often even negative at low temperatures, then rises, approaching a constant value at high temperatures. On the other hand, for less hydrophilic solutes and for solutions with high concentrations, a decrease of $S_{T}$ with temperature has been observed [3,9,12]. This has been connected to reduced interactions between solute and solvent, through reduced attraction between the components.

It turns out that the hydrophilicity of the solute (or hydrophobicity, if the affinity is small) plays an important role in the thermophoresis of aqueous systems. One of the most frequently used parameters to describe hydrophilicity is the 1-octanol partition coefficient *P*. It was found that temperature dependence of $S_{T}$ correlates with the logP of the solute [9,12]. For lower logP-values, a stronger change of the Soret coefficient with temperature is observed. This might indicate that a larger number of water molecules connect to the solute molecule via hydrogen bonds. As the hydrogen bonds get weaker with increasing temperature, we expect a larger change in the hydration layer, if more water molecules are bound to the solute. As the Soret effect is an interfacial effect [14,15,16], we expect therefore a larger change of the thermophoretic behavior, if more water molecules form hydrogen bonds with the solute.

Thermophoresis depends also on the mass difference between solute and solvent. There are numerous studies on isotope effects especially in non-polar [17,18,19], but also in polar mixtures [20]. For non-polar systems, the isotopic Soret coefficient $S_{T}^{i}$ with the contribution resulting from the differences in molecular mass and moment of inertia between solute and solvent has been introduced as

(2) $$S_{T}^{i}=a_{M}\Delta M+b_{I}\Delta I$$

with numerical factors $a_{M}$ and $b_{I}$ [18].

There are now a variety of validated methods that allow quantitative studies of the effect in binary [21] and ternary systems [22,23]. All biological systems of interest are multicomponent systems that typically contain buffer ingredients or salts to stabilize the proteins in solution. In order to investigate those systems, microscopic methods with fluorescent detection are used, for example to determine the local protein concentration. Either a fluorescent label is attached or the inherent fluorescence of the molecule of interest is detected [24]. While these methods are capable of determining binding constants, the temperature profile is often not known very accurately. In most cases, the temperature is determined from the temperature dependence of the fluorescent intensity of a dye [25,26]. The temperature resolution is typically only of the order of a 1% change of the fluorescent intensity per Kelvin [26]. This low sensitivity as well as the complex and unpredictable photophysical behavior make it difficult to obtain reliable temperature measurements.

We use streptavidin (STV) and the ligand biotin (B) as a model system to measure the thermophoretic change between the free STV and its ligand-bound state (STV+B). STV in its natural state is a homotetramer, which binds four biotins. STV is known for its extremely high binding affinity to biotin, with the dissociation constant in the range of $K_{d}\approx 4\times 10^{-14}$ M [27,28,29].

The measurements discussed in this work are motivated by the question if thermophoretic data could be used to attain information about changes in the hydration shell upon protein–ligand binding. In order to gain a deeper understanding, we compare our thermophoretic data with recent quasi-elastic neutron scattering (QENS) measurements [30]. Neutron scattering experiments probe the internal dynamics of the protein and can be used to determine the entropic change of the protein in ligand-binding reactions or in protein folding [30,31], while thermophoresis is more sensitive to the hydration layer and the accompanying entropic changes of the surrounding water molecules. In general, structural differences of a protein dissolved in D${}_{2}$O vs. H${}_{2}$O cannot be excluded. However, for STV, both small angle X-ray scattering curves in H${}_{2}$O [30] and small angle neutron scattering data in D${}_{2}$O [32] agree within experimental errors with calculated scattering curves based on high resolution crystal structures of free STV and its ligand-bound state (STV + B). Nevertheless, this observation does not exclude differences in the interactions of the protein with H${}_{2}$O and D${}_{2}$O, respectively.

## 2. Experimental Section

### 2.1. Thermal Diffusion Forced Rayleigh Scattering

**Figure 1:**
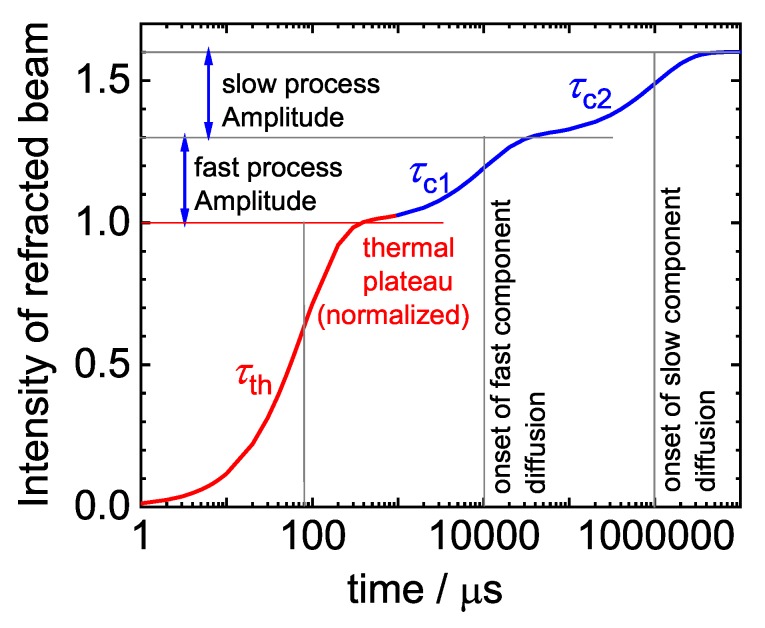
Schematic diagram of the heterodyne intensity of the diffracted beam for a ternary mixture against time. We can differentiate the thermal (red) and the concentration (blue) part of the signal. The three time constants $\tau_{\mathrm{th}}$, $\tau_{c1}$ and $\tau_{c2}$ describe the equilibration of the temperature, the solvent and the solute diffusion according to Equation (5). For a binary mixture we would observe only one mode of the concentration signal (blue) as described in Equation (4).

Thermodiffusion of STV was measured by infrared thermal diffusion forced Rayleigh scattering (IR-TDFRS) [33,34]. This method uses the interference grating of two infrared laser beams (λ=980 nm) to generate a temperature grating inside an aqueous sample due to the inherent absorbtion of water in that wavelength range [35]. A third laser beam is refracted by this grating and the heterodyne intensity of the diffracted beam is measured as a function of time. This intensity is proportional to the refractive index contrast of the grating, showing a fast initial rise over time due to the thermal gradient, then a slower change of intensity due to diffusion of the solute along the temperature gradient (cf. Figure 1). The heterodyne scattering intensity $\zeta_{het}\left(t\right)$ of the read-out beam is fitted by two exponential functions

(3) $$\begin{array}{c} \zeta_{\mathrm{het}}\left(t\right)=1-\exp\left(-\frac{t}{\tau_{\mathrm{th}}}\right)-A{\left(\tau_{c}-\tau_{\mathrm{th}}\right)}^{-1}\;\;\\ \times \left\{\tau_{c}\left[1-\exp\left(-\frac{t}{\tau_{c}}\right)\right]-\tau_{\mathrm{th}}\left[1-\exp\left(-\frac{t}{\tau_{\mathrm{th}}}\right)\right]\right\}, \end{array}$$

with $\tau_{\mathrm{th}}$ the equilibrium time of the thermal grating, with $\tau_{c}=1/\left(q^{2}D\right)$ the time constant of the ordinary translational diffusion, *D* the diffusion coefficient and *q* the magnitude of the grating vector of the optical grating with the fringe spacing d=2π/q. The Soret coefficient $S_{T}$ can be calculated from the amplitude *A*, if the concentration *c* and the so-called contrast factors, the change of refractive index with temperature and concentration, ${\left(\partial n/\partial T\right)}_{c,p}$ and ${\left(\partial n/\partial c\right)}_{T,p}$, are known:

(4) $$A={\left(\frac{\partial n}{\partial c}\right)}_{p,T}{\left(\frac{\partial n}{\partial T}\right)}_{p,c}^{-1}S_{T}\;c\left(1-c\right).$$

The thermal process is 3–4 orders of magnitude faster than the diffusive motion of the solute, so that the two processes can be separated.

Ternary mixtures consisting of a high molar mass solute at low concentration in a solvent mixture can also be analyzed by TDFRS [22,36]. In such a ternary mixture, the concentration signal contains a fast and a slow mode resulting from the solvent molecules and the slower diffusing solute. The heterodyne scattering intensity $\zeta_{het}\left(t\right)$ of the read-out beam can then be described by a sum of three exponential functions

(5) $$\begin{array}{c} \zeta_{\mathrm{het}}\left(t\right)=1-\exp\left(-\frac{t}{\tau_{\mathrm{th}}}\right)-A_{1}{\left(\tau_{c1}-\tau_{\mathrm{th}}\right)}^{-1}\;\;\;\;\\ \times \left\{\tau_{c1}\left[1-\exp\left(-\frac{t}{\tau_{c1}}\right)\right]-\tau_{\mathrm{th}}\left[1-\exp\left(-\frac{t}{\tau_{\mathrm{th}}}\right)\right]\right\}\\ -A_{2}{\left(\tau_{c2}-\tau_{\mathrm{th}}\right)}^{-1}\\ \times \left\{\tau_{c2}\left[1-\exp\left(-\frac{t}{\tau_{c2}}\right)\right]-\tau_{\mathrm{th}}\left[1-\exp\left(-\frac{t}{\tau_{\mathrm{th}}}\right)\right]\right\} \end{array}$$

with $\tau_{c1}$ and $\tau_{c2}$ the time constants of the solvent and the solute diffusion, respectively. Note that this approach neglects the off-diagonal elements of the diffusion matrix.

### 2.2. Sample Preparation

The STV was obtained commercially (ProSpec-Tany TechnoGene Ltd., Ness-Ziona, Israel, catalogue number pro-791-c). The molecular weight M=53.1 kDa was determined by mass spectrometry. The protein powder was desalted using PD-10 desalting columns (GE Healthcare, Chicago, IL, USA). For the TDFRS samples, the lyophilized STV was dissolved in buffer (25 mM Tris-HCl, 120 mM NaCl, 5 mM KCl, 3 mM MgCl${}_{2}$, pH = 7.4) that was filtered (0.2μm) to remove dust particles. The buffer was prepared with 100% H${}_{2}$O or contained a specified amount of D${}_{2}$O (up to 50%). The mixture was centrifuged to remove dust particles (3 min, 6000 rpm) and the clear solution filled into an optical quartz cell (Hellma) with an optical path length of 0.2 mm. The STV concentration was checked by using Nano-Drop 2000c (Thermo Scientific, Waltham, MA, USA, $\epsilon_{1\%}$ = 31.29 at 280 nm). The UV absorption-based concentration was ≈22% smaller than the gravimetrically determined one.

The buffer has an ionic strength of I=0.16 M. The corresponding high salt concentration results in a Debye length of $\kappa^{-1}\approx 0.8$ nm. This ensures that electrostatic interactions between colloidal surface and surrounding solvent are very short-ranged [37].

### 2.3. Contrast Factor Measurement

The change of refractive index with mass concentration ${\left(\partial n/\partial c\right)}_{p,T}$ was measured by a refractometer (RXA 156, Anton Paar, Ostfildern, Germany, accuracy 0.00002 nD, ΔT = ±0.03 K). The refractometer uses a wavelength of 589.3 nm (sodium line), which is shorter than the wavelength of the read-out beam in our IR-TDFRS setup (HeNe-laser, 632.8 nm). This causes a small systematic error in the refractive index increment on the order of 0.5–1% [38,39]. Refractive indices were measured for at least four concentrations at five different temperatures (10–50 °C) for each system. The concentration dependence of *n* was linearly fitted to derive the slope ${\left(\partial n/\partial c\right)}_{p,T}$ for all measured temperatures. For intermediate temperatures, the data were interpolated. The refractive index increments with temperature ${\left(\partial n/\partial T\right)}_{p,c}$ were measured interferometrically [40]. Measurements were performed for all systems investigated by IR-TDFRS, across the temperature range of 10–50 °C and with a heating rate of 2 mK/sec or less.

### 2.4. Evaluation

**Figure 2:**
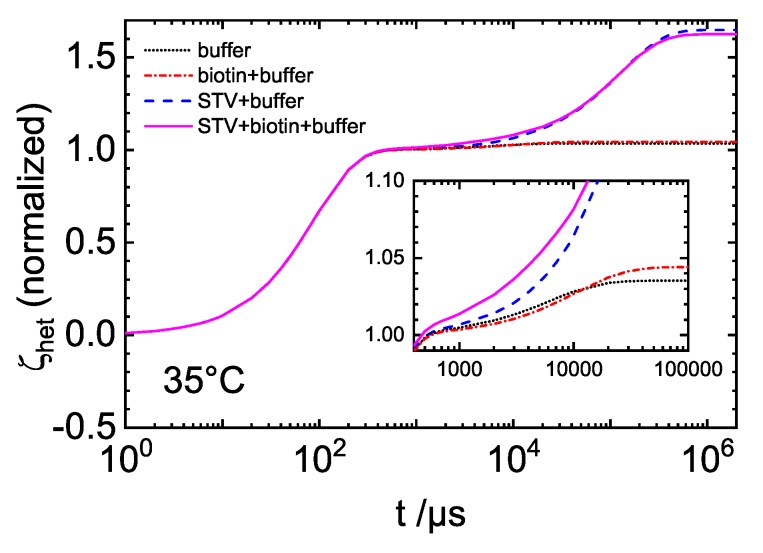
Normalized heterodyne diffraction intensities as function of time for various solutions: buffer (black dotted line), B + buffer (red dash-dotted line), STV + buffer (blue dashed line) and STV + B-buffer (magenta solid line).

The investigated systems are in fact multi-component systems, with the buffer containing five types of ions. Figure 2 shows an example of the normalized heterodyne signals $\zeta_{\mathrm{het}}$ for measurements of buffer, biotin(B)+buffer, STV+buffer and STV+B+buffer solutions at 35 °C. The buffer components and biotin, covering a mass range of 23 to 244 g/mol, cannot be distinguished from each other and all contribute to the ‘fast component’ signal (first shoulder after the thermal plateau in Figure 2). Likewise, the free STV and its complexes with biotin (∼53 kDa) are observed as one signal (‘slow process’, last plateau in Figure 2). If we compare the buffer and buffer with biotin signals, we see that the biotin+buffer amplitude is only roughly 20% larger than the buffer signal. This is a consequence of the low biotin amount compared to the salt content and the higher refractive index contrast of the salt components. While the signal of the protein and its complex can be well separated from the faster components, the two fast processes cannot be distinguished.

On the other hand, the signal of the slow process is well separated and its amplitude and time constant can be determined with an uncertainty of 1–2% (cf. Appendix A Table A1). Therefore, the determination of the slower compounds is reliable.

**Figure 3:**
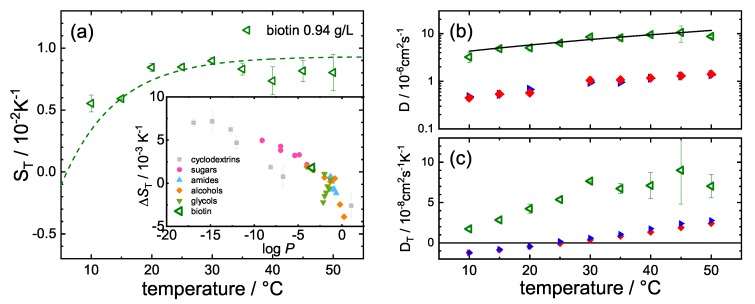
(**a**) Soret coefficient $S_{T}$ of biotin against temperature. The inset shows $\Delta S_{T}$ versus logP for biotin and other substance classes. (**b**) The measured diffusion coefficient *D* of biotin (green triangles) in comparison with the calculated diffusion coefficient (solid black line). *D* of STV (blue triangles) and STV + B (red diamonds) is roughly one order of magnitude slower. (**c**) The measured thermal diffusion coefficient $D_{T}$ of biotin (green triangles), of STV (blue triangles) and of STV + B (red diamonds) against temperature.

**Figure A1:**
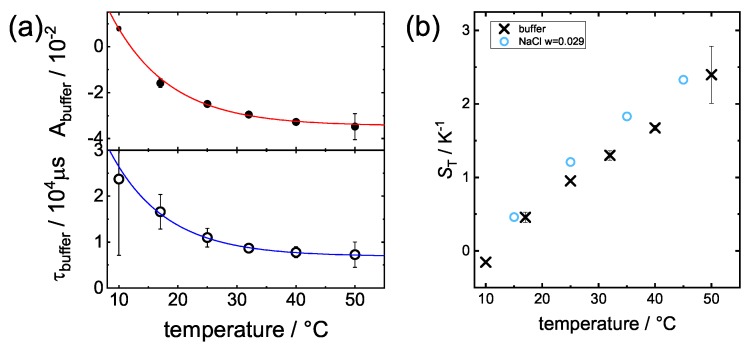
(**a**) Amplitude, $A_{\mathrm{buffer}}$, of the concentration part of the normalized heterodyne signal (cf. Equation (4)) of the buffer solution and (**b**) the corresponding time constant, $\tau_{\mathrm{buffer}}$, as function of temperature. The lines correspond to an exponential fit of the data. Note, that the solutions contain 120 mM NaCl corresponding to a weight fraction of w=0.007.

The question arises as to whether we can also obtain some meaningful thermophoretic data for biotin in the buffer solution. In order to extract coefficients for biotin, we fitted the heterodyne signal for the biotin+buffer solution using a double exponential function with one fixed exponential mode. Here, we used the amplitude and times determined for the buffer (see Appendix A Figure A1). The resulting Soret coefficient $S_{T}$, diffusion coefficient *D*, and thermal diffusion coefficient $D_{T}$ of biotin as a function of temperature are shown in Figure 3. Especially at high temperatures, the error bars are typically below 10%.

Note that we assumed that the thermophoretic behavior of buffer compounds and biotin are incremental. In order to check this hypothesis for consistency, we calculated $\Delta S_{T}=S_{T}\left(50\;{}^{\circ}C\right)-S_{T}\left(20\;{}^{\circ}C\right)$ of biotin and compared it with the other investigated systems [1,9]. Note that, at pH 7.4, biotin is negatively charged leading to logP=3.4 [41]. Unfortunately, there are to our best knowledge no biotin diffusion coefficients reported in the literature; therefore, we estimated the diffusion coefficient using the Stokes–Einstein relation $D=k_{B}T/\left(6\pi \eta R\right)$ with the Boltzmann constant $k_{B}$ and the dynamic viscosity, η, of the buffer. We used the viscosity of pure water [42] as the buffer consists mainly of water. The hydrodynamic radius *R* we estimated from the van der Waals volume $V_{\mathrm{vdW}}=213$ Å${}^{3}$ of biotin [41] assuming a sphere. The calculated diffusion coefficients (solid black line in Figure 3b) agree within the error bars with the determined diffusion coefficients.

## 3. Results

### 3.1. Change upon Ligand Binding

**Figure 4:**
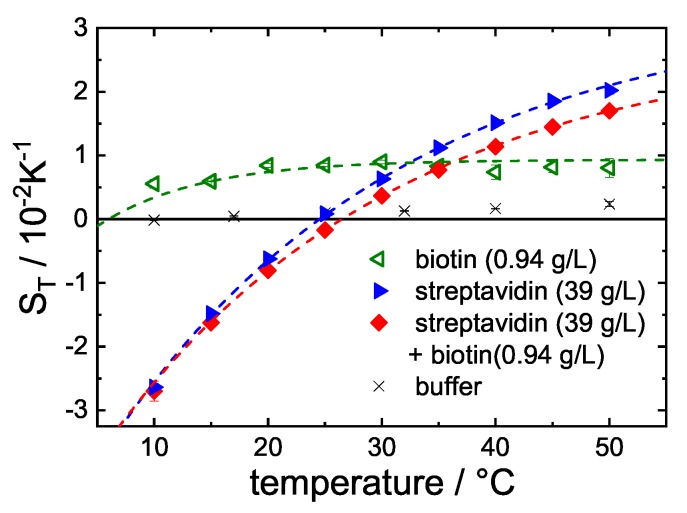
Soret coefficient $S_{T}$ against temperature. Shown are biotin (green), STV (blue) and STV + B (red), all in H${}_{2}$O-buffer. The signal of the buffer itself (black crosses) is about one order of magnitude smaller compared to biotin. Note, the slight excess of biotin (cf. Appendix A Table A2).

As expected, a significant difference of the Soret coefficient $S_{T}$ can be observed between free STV and the STV + B complex (Figure 4, blue triangles and red diamonds, respectively). As shown in Figure 3b, the ligand binding has no influence on diffusion. Diffusion of the buffer components and biotin is significantly faster than that of STV (53 kDa), so that the set-in of diffusion for the smaller components and the protein can be identified separately in the measured intensity signal.

In contrast, the thermal diffusion (cf. Figure 3c) and Soret coefficient (cf. Figure 4) of the complex are reduced compared to the free protein. The difference between $D_{T}$ and $S_{T}$ of the free protein and the complex increases with increasing temperature and almost vanishes below room temperature. One possible explanation is that the free STV is already relatively stiff below room temperature, so that the binding of biotin has a weaker effect than at higher temperatures. Figure 4 also shows $S_{T}$ against *T* of the buffer alone (black crosses) and biotin in buffer (green triangles). It turns out that the Soret coefficient of the buffer is of the same order of magnitude as the major component of the buffer, i.e., NaCl (cf. Appendix A Figure A1b).

As mentioned in the Introduction, the variation of $S_{T}$ with temperature is more pronounced, if the solute forms more hydrogen bonds with water. This implies that the free STV is slightly more hydrophilic than the complex STV+B. Furthermore, it is known that the high affinity between STV and B is characterized by an extensive hydrogen bond network [43] inside the binding pockets of the tetrameric STV. According to Hyre et al. [44], five of the hydrogen bond sites are deep in the pocket, so that they are no longer accessible for water once the biotin binds. This might be one reason for the slightly less hydrophilic character of the STV+B complex. In addition, various unmethylated cyclodextrins (CDs) and their corresponding complexes with acetylsalicylic acid (ASA) showed a similar behavior. The temperature dependence of the CDs was more pronounced than that of the CD+ASA complex, indicating a slightly less hydrophilic character of the complex [45]. Another reason for the slightly lower hydrophilicity of the complex might be the higher flexibility of the STV compared to STV+B, which was recently investigated by quasi elastic neutron scattering (QENS) [30]. When Liese et al. [46] investigated stretched (stiff) and flexible poly(ethylene glycol) (PEG) chains, they found that entropic hydration effects compensate almost completely the chain conformational entropy, or, in other words, the water molecules in the hydration layer of the stiff stretched PEG form fewer hydrogen bonds compared to the flexible PEG coil. A similar argument might also hold for the STV + B complex.

### 3.2. Influence of Stoichiometry

**Figure 5:**
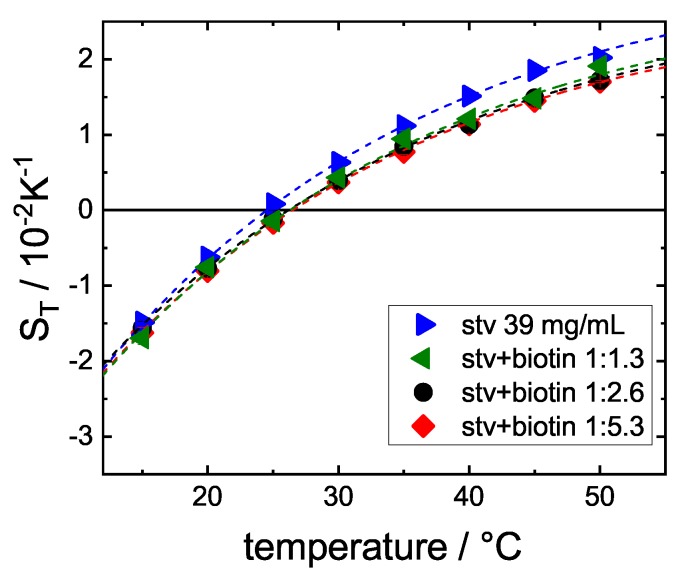
Soret coefficient $S_{T}$ against temperature for STV (39 mg/mL) with different stoichiometries of biotin. The results for STV without biotin (blue) and for the ratio 1:5.3 (red) are reproduced from Figure 4 in pale blue and red for easy comparison. The exact concentrations are summarized in Table A2 of the Appendix A.

In the next set of experiments, the biotin concentration was varied. STV is a tetramer and each monomer is able to bind one biotin. Here, the ratio STV+B was lowered below 1:4, so that STV is not fully saturated with ligand. The biotin concentrations used are summarized in the Appendix A in Table A2. Figure 5 shows that, already at a STV+B ratio of ∼1:1, a significant change of the thermodiffusion behavior can be observed. Increasing the biotin concentration further only leads to a relatively small decrease in $S_{T}$. The fact that the dependence on biotin concentration is not linear is an indication of cooperative binding [28,30] between STV and biotin: a conformational change is already (partly) induced when only one monomer has bound to biotin. Such cooperativity effect for the STV and biotin has been observed in electrophoresis experiments [28].

### 3.3. Influence of D${}_{2}$O

The recent neutron scattering experiments are carried out in D${}_{2}$O [30]. However, for IR-TDFRS, we use infrared light with a wavelength of 980 nm to generate the temperature gradient inside the sample. While H${}_{2}$O absorbs at that wavelength, D${}_{2}$O does not, so that it is not possible to carry out experiments in pure D${}_{2}$O. Up to a D${}_{2}$O-concentration of 50% measurements is possible, even though it has to be noted that the refractive index contrast is lower due to the weaker temperature gradient and the noise of the thermal plateau increases from 1% to 10–20%.

**Figure 6:**
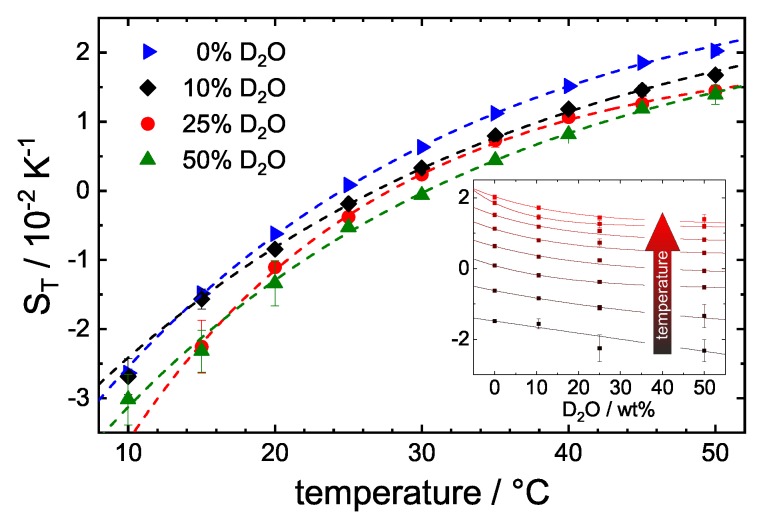
Soret coefficient $S_{T}$ against temperature for STV (39 mg/mL) in buffer solutions with varying percentages of D${}_{2}$O. The inset shows $S_{T}$ against the percentages of D${}_{2}$O for different temperatures.

Figure 6 shows $S_{T}$ against temperature for varying D${}_{2}$O-contents. The inset of Figure 6 shows a reduction of $S_{T}$ with increasing D${}_{2}$O content. Due to the decreasing signal-to-noise ratio, we can not identify a significant change in the temperature sensitivity of $S_{T}$. In the literature, there is some evidence suggesting that the deuterium bond is slightly stronger [47,48,49]. For instance, the frequency of the vibrational mode decreases by roughly 3% due to deuterium substitution. Therefore, one could expect a more pronounced temperature sensitivity of $S_{T}$ with increasing heavy water content. Apparently, the change is too weak or of the same order as the uncertainty of $S_{T}$, so that we are unable to detect it. On the other hand, it is not certain that H${}_{2}$O and D${}_{2}$O are homogenically distributed between bulk and hydration layer around the protein in the investigated mixture. An enhanced hydrophobic effect would imply that a higher D${}_{2}$O concentration in bulk and more H${}_{2}$O in direct contact with the protein might be energetically prefereable. Since we were only able to investigate solutions up to 50% D${}_{2}$O concentration, it is also possible that there is not yet a strong change in the composition of the hydration layer.

According to Cioni and Strambini [50], the addition of D${}_{2}$O might lead to a stiffening of the protein and can have a similar effect as biotin. On the other hand, a comparison of recent small angle x-ray scattering (SAXS) and QENS experiments shows that the structure of STV is not affected by exchanging H${}_{2}$O with D${}_{2}$O [30], which would suggest that the changes observed in the TDFRS are mainly caused by a change in the mass difference between protein and solvent. As the mass difference between STV and water is larger compared to heavy water, we expect, according to Equation (2), a decrease of $S_{T}$ with increasing heavy water content [18,20]. This is in accordance with the experimental observations.

**Figure A2:**
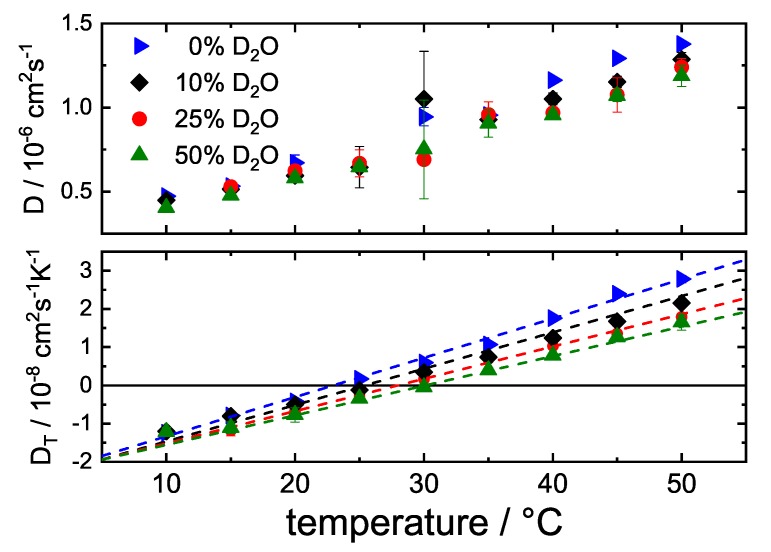
Diffusion coefficient *D* and thermal diffusion coefficient $D_{T}$ against temperature for streptavidin (39 mg/mL) in buffer solutions with varying percentages of D${}_{2}$O.

The corresponding diffusion data are displayed in the Appendix A in Figure A2. The diffusion decreases slightly due to the higher viscosity of $D_{2}$O. The extrapolated diffusion coefficient of STV in a 100% D${}_{2}$O buffer at 20 °C is D≈5.8${\overset{˚}{A}}^{2}/\mathrm{ns}$, which agrees well with the interpolated diffusion coefficient found by DLS at 40 mg/mL with D≈5.7${\overset{˚}{A}}^{2}/\mathrm{ns}$ [30]. In water, the diffusion coefficient of STV at 20 °C measured by TDFRS is D=6.7±0.5${\overset{˚}{A}}^{2}/\mathrm{ns}$ (39 mg/mL) and agrees with the literature value of D=6.2${\overset{˚}{A}}^{2}/\mathrm{ns}$ [51].

The temperature dependence of $D_{T}$ decreases with increasing D${}_{2}$O content. Additionally, the differences in $D_{T}$ with varying D${}_{2}$O content are larger at higher temperatures. This is probably a consequence of the difference in the ratio of thermal expansion coefficient α over viscosity η: the difference of the ratio between light and heavy water increases with increasing temperature [52,53]. Earlier studies have shown that the thermal diffusion coefficient is proportional to the ratio α/η [54,55]. This implies that the thermal diffusion coefficient becomes larger for systems which show a larger thermal expansion coefficient and which are less viscous.

## 4. Discussion

The results show that, while TDFRS is a reliable tool to characterize the thermodiffusion behavior of protein systems, the highly complex nature of these systems necessitates careful and systematic measurements. Nevertheless, the results of the fairly large proteins and protein complexes are insensitive to the contribution of buffer components and ligand. In contrast, the separation of the ligand contribution from the buffer is more difficult. For this particular system, we were able to obtain reasonable results for the ligand biotin, by using the amplitude and the time constant determined for the pure buffer. The temperature dependence of the Soret coefficient of biotin lies on the logP master curve [1], and the measured diffusion coefficients correspond to the calculated van der Waals volumes. Whether this is generally true needs to be investigated more systematically for a larger number of ligands and salts. One could, for instance, expect deviations for buffers containing salts which influence the hydrogen bond network more strongly [56].

The temperature dependence of the Soret coefficient of the free protein compared to the complex reveals that the protein-complex is slightly less hydrophilic. One reason is that several hydrogen bond sites are deep in the pocket and no longer accessible once biotin has bound to STV [30,44]. Additionally, the complex is stiffer compared to the free STV, so that the conformational change of the protein increases the hydration degrees of freedom [46]. An increase of the entropy of the hydration layer has been hypothesized, which almost compensates the decrease of the conformational entropy [30].

Our investigations illustrate that thermodiffusion and its temperature dependence are highly sensitive to and can provide valuable information about changes in the hydration layer of protein systems upon ligand binding. While many questions about the origin and exact mechanisms of these changes can not be answered on the basis of the behavior of only one protein–ligand system, similar measurements of similar systems might provide a clearer picture and lead to valuable insights into the interplay of conformational changes and hydration in the future.

Additionally, there is a need for the development of new methods for multi component systems, which are capable of monitoring the protein and its complex individually. While the faster components can be easily separated, this will not be the case for the free protein and its complex. This is an important issue for systems with lower binding constants compared to STV+B as those systems can no longer be treated as pseudo-binaries, so the development of multicomponent theories for polar systems would be desirable. In order to investigate the influence of D${}_{2}$O, it would be worthwhile to perform experiments with a beam deflection method using direct heating instead of optical heating [57]. This way, it would be possible to pinpoint the differences between normal and heavy water.

## Figures and Tables

**Figure A3:**
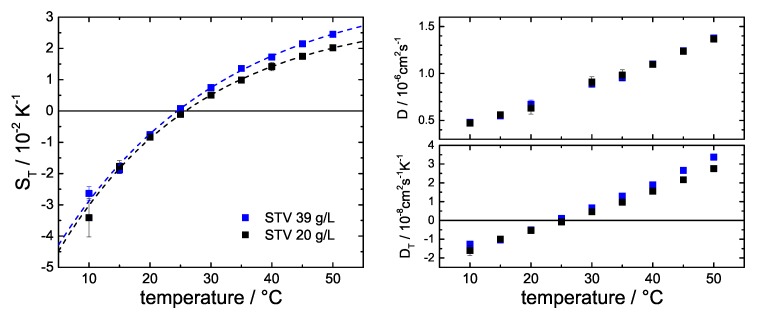
Soret coefficient $S_{T}$, diffusion coefficient *D* and thermal diffusion coefficient $D_{T}$ against temperature for streptavidin 39 mg/mL and 20 mg/mL.

## Abbreviations

The following abbreviations are used in this manuscript:

TDFRS	Thermal Diffusion Forced Rayleigh Scattering
QENS	Quasi-Elastic Neutron Scattering
STV	Streptavidin
B	Biotin

## Appendix A. Evaluation

In Section 2.4, Figure 2 shows typical normalized heterodyne signals $\zeta_{\mathrm{het}}$ for measurements of buffer, biotin(B)+buffer, STV+buffer and STV+B+buffer solutions at 35 °C. While the signal of the protein and its complex can be well separated from the faster components, the two fast processes cannot be distinguished. This becomes also obvious if we look at Table A1. As an example, the corresponding amplitudes and time constants for various solutions are summarized. Note that the numbers in bold red have been fixed during the fitting process. For the STV and the B+STV, the determined amplitudes $A_{\mathrm{STV}}$ and time constants $\tau_{\mathrm{STV}}$ agree with 1–2%, while the amplitudes of the fast component vary by 20–30%.

**Table A1:** As an example the amplitudes and time constants for buffer, B + buffer, STV + Buffer and STV + B-buffer solutions at 35 °C. The parameters in bold print have been fixed during the fitting process.

Sample	$A_{\mathrm{buffer}}$	$\tau_{\mathrm{buffer}}$	$A_{B}$	$\tau_{B}$	$A_{\mathrm{STV}}$	$\tau_{\mathrm{STV}}$
		μs		μs		μs
buffer	−0.03521	6129				
0.044	10570					
	−0.01206	**6129**	−0.03256	**10,570**		
	−0.01429	**6129**	−0.03048	10,890		
	−0.00675	3034	−0.03917	10,200		
STV					−0.6232	127,600
	−0.03087	**6129**			−0.6228	**127,600**
	−0.03724	**6129**			−0.62	128,900
	−0.03382	11910			−0.6146	129,700
STV + B	−0.03737	3153			−0.5879	122,000
	−0.02486	**6129**			−0.5737	122,100
			−0.01411	**10,570**	−0.5636	122,100

In order to extract some reasonable Soret coefficients for biotin, we fixed amplitude and times of the buffer solution to the interpolated values at the measurement temperature shown in Figure A1a. The resulting Soret coefficients of biotin have standard deviation values of typically 10–20%. A direct measurement of biotin in water is not possible due to its low solubility of 0.22g/L. We can also analyze the amplitudes of the buffer itself. The determined Soret coefficient are of the order of the Soret coefficients of the major component NaCl. Note that the NaCl weight fraction in the buffer is 0.006, while the total weight fraction of all solute components is 0.010.

### Appendix A.1. Influence of Stoichiometry

The biotin concentrations used to study the influence of the stoichiometry are shown in Table A2. There is always a slight excess amount of biotin.

**Table A2:** Biotin concentrations corresponding to the molar streptavidin:biotin ratios. Streptavidin concentration was 39±1 mg/mL ($7.3\pm 0.2\times 10^{-7}$ mol/mL) for all samples.

STV:Biotin	Biotin mg/mL	Concentration mol/mL
1:1.3	0.24	9.8 $\times \;10^{-7}$
1:2.6	0.48	19.6 $\times \;10^{-7}$
1:5.3	0.94	38.5 $\times \;10^{-7}$

### Appendix A.2. Influence of D_2_O

The diffusion data shown in Figure A2 correspond to the Soret coefficients shown in Figure 6 in Section 3.3. We can see the trend induced by an exchange of H${}_{2}$O with D${}_{2}$O. The diffusion decreases slightly due to the higher viscosity of D${}_{2}$O. The data are quite noisy, but we can conclude that, with increasing D${}_{2}$O-content, the diffusion slows down.

The temperature dependence of $D_{T}$ decreases with increasing D${}_{2}$O content. The differences in $D_{T}$ with varying D${}_{2}$O content are larger at higher temperatures.

### Appendix A.3. Comparison of Two Concentrations

We also looked briefly into the temperature dependence of $S_{T}$, $D_{T}$, and *D* at two protein concentrations. A measurement at lower STV concentration of 30 g/L shows that, while the Fickian diffusion does not seem affected, the thermal diffusion coefficient $D_{T}$ is reduced for the lower concentration. This reduction of $D_{T}$ is more pronounced at higher temperatures. In contrast to the studies of uncharged systems [9], we observe a stronger temperature dependence for the higher concentration. This might be due to the charged components in the buffer, but further systematic studies are required to solve this issue.
